# Examining Family Atmosphere and School Climate Within Psychology of Working Theory Among Chinese Rural College Students

**DOI:** 10.3390/bs14121151

**Published:** 2024-12-02

**Authors:** Lu Hai, Xiaohong Bao, Yang Wang, Mengxiao Zhang, Man Shu

**Affiliations:** 1 School of Education, Minzu University of China, Beijing 100081, China; 2011003@muc.edu.cn (L.H.); 24400211@muc.edu.cn (Y.W.); 2 College of Special Education, Changchun University, Changchun 130000, China; 3 Faculty of Education, Northeast Normal University, Changchun 130024, China; zhangmx100@nenu.edu.cn; 4 Department of Sociology, Peking University, Beijing 100871, China; 2401111177@stu.pku.edu.cn

**Keywords:** psychology of working theory, family atmosphere, school climate, Chinese rural college students

## Abstract

The employment situation for college students has worsened due to the increase in the number of graduates and the impact of the COVID-19 pandemic on the economy. Consequently, the pressure to find employment has also increased, particularly for rural college students. Drawing on the Psychology of Working Theory (PWT), the present study was performed to examine the applicability of some proposed pathways in the PWT and test the relationships between family atmosphere and school climate and work volition, career adaptability, and future decent work perception within 946 rural college students (243 men and 703 women; Mage = 19.86, SD = 1.48). The study employed a cross-sectional research design, and structural equation modeling (SEM) was used. Results indicated that family atmosphere and school climate significantly positively predict work volition, career adaptability and future decent work perception. Work volition significantly predicts career adaptability and future decent work perception. Moreover, our findings supported the mediating effect of work volition, indicating that family atmosphere and school climate increased career adaptability and future decent work perception by improving work volition. Although this study has limitations, it informs future studies by highlighting the important role of family atmosphere, school climate, and work volition.

## 1. Introduction

With the rapid development of Chinese society, more and more young people have opportunities to receive higher education; however, it has become more difficult for them to enter some desirable enterprises [1]. Differing from many theories, as pertains to career acquisition and development issues, the Psychology of Working Theory (PWT) [2] shifted attention to the contextual factors of individuals’ access to decent and equitable work and focused on disadvantaged groups (e.g., gender and sexual minorities). Rural college students face various barriers such as a lack of stable economic sources and necessary material support and weak psychological quality in job hunting [3]. Therefore, it may be difficult for them to secure decent work in the future. Studies based on the PWT have fully examined the relationship between economic constraints, work volition, career adaptability, and future decent work perception, but there are still knowledge gaps. Previous studies rarely considered the influence of family and school factors on individual occupation within the PWT, and did not focus on rural college students. Therefore, considering the cultural tradition of emphasizing environment and collectivism in China and the fact that college students mainly live in school and with family, this paper will take family atmosphere and school climate as the second and third contextual factors, which parallel with economic constraints, and examine the complex relationship among these three variables and the work volition, career adaptability, and future decent work perception of rural college students, which will further expand the application of the theory and enrich the results of current academic research.

### 1.1. The Relationships Between Variables of PWT

Based on the related studies in multicultural psychology, vocational psychology, and the sociology of work, especially from the Psychology of Working Framework (PWF) [4,5,6], Duffy et al. proposed the PWT [2]. The PWT describes how contextual factors predict an individual’s vocational outcomes, especially those near or in poverty, facing discrimination and marginalization, or facing challenging work-based transitions [2]. Decent work as a vocational outcome is the central variable; the predictors of securing decent work are economic restraints and marginalization; the mediator variables are work volition and career adaptability, and the moderator variables are proactive personality, critical consciousness, social support, and economic conditions, while the outcomes of decent work are survival needs, social connection needs, self-determination needs, and the outcomes of need satisfaction are work fulfillment and well-being [2].

Taking into account the focus and target population of this study, the upcoming literature review section will primarily examine the relationships between economic constraints, work volition, career adaptability, and decent work, as identified in previous research articles focusing on university students.

Economic constraints refers to the limited economic resources and social or capital resources that represent a critical barrier to securing decent jobs [2]. Allan et al., Kim et al., and Hai et al. have found the effect of economic constraints on work volition [7,8,9], the perceived capacity to make occupational choices despite constraints [10]. Therefore, taking into account authors Allan, Kim, and Hai, we suggest that economic constraints significantly negatively predict work volition (Hypothesis 1). Additionally, Autin et al. found the effect of economic constraints on career adaptability [11]: the capacity to plan work, adjust to work responsibilities, and recover when work-related adversity arises, which consists of four components, which are concern, control, curiosity, and confidence [12]. Based on Autin and colleagues’ studies, we suggest that economic constraints negatively predict career adaptability (Hypothesis 2). In addition, Ma et al. found the effect of economic constraints on future decent work perceptions [13]. Decent work refers to work that is provided with adequate compensation, free time and rest, health care, protection from physical and psychological harm, and which complements family and social values. Referring to author Ma, we suggest that economic constraints negatively predict future decent work perceptions (Hypothesis 3). For unemployed college students, we measured their perceptions of future decent work, which seems more suitable for them. This approach is consistent with previous research practices [14]. The primary reason for the relationship between economic constraints and work volition, career adaptability, and future decent work perceptions may be that economic deprivation may specifically restrict students’ ability to engage in career-building activities in college, such as unpaid internships and extracurricular activities that may allow students to have greater work choice in their future, which may result in students having fewer job options [7] and lower levels of career preparedness.

Moreover, a few studies have investigated the role of work volition on career adaptability and found that work volition has a manifest impact on career adaptability. For example, Ma et al., Wei et al., and Duffy et al. confirmed that those who have higher work volition would feel more adaptable in their career lives [13,14,15]. Taking into account Ma, Wei, and Duffy, we suggest that work volition positively predicts career adaptability (Hypothesis 4). Additionally, we suggest that work volition positively predicts future decent work perception (Hypothesis 5), based on the studies of Ma et al., Wei et al., and Duffy et al.

For Hypothesis 6, that career adaptability positively significantly predicts future decent work perception, we base ourselves on the studies of authors Ma et al., Wei et al., Guo et al., and Kim et al. who suggested that career adaptability could directly predict perceptions of future decent work [13,14,16,17]. This may be because, when individuals are well prepared for their future careers, they are more likely to discover quality job opportunities and successfully pass the evaluation process, which ultimately leads to decent work.

Moreover, since work volition is affected by economic constraints as well as career adaptability, and future decent work perception was also affected by work volition, as mentioned above, we assume that work volition mediates the association between economic constraints and career adaptability (Hypothesis 7) and the association between economic constraints and future decent work perception (Hypothesis 8). Since career adaptability is affected by economic constraints and work volition, and future decent work perception was also affected by career adaptability, as mentioned above, we assume that career adaptability mediates the association between economic constraints and future decent work perception (Hypothesis 9) and the association between work volition and future decent work perception (Hypothesis10).

### 1.2. The Relationship Between Family Atmosphere and School Climate with Variables of PWT

According to ecological systems theory [18], families and schools are college students’ mesosystem. The family college students have been living in since birth provides them with ample support in terms of finances and daily life. School is the place where they spend most of their time enhancing their knowledge and cognitive abilities. The influence of family and school on individuals is profound, particularly in China, where a strong emphasis is placed on collectivist culture. For example, scholars have suggested that school climate is an essential factor relating to students’ future career development [19,20]. Therefore, this article will focus on investigating the relationship between family atmosphere and school climate with the variables mentioned above in the PWT.

Family atmosphere refers to the family’s internal psychological process, family members’ behavior, communication, and the interaction between the family and the external environment [21,22]. Previous studies found that family atmosphere significantly affects work volition. For example, Lustig and Xu indicated that higher levels of family cohesion were associated with lower levels of decision-making confusion, which indicated that the family atmosphere might predict an individual’s effective work choices [23]. Therefore, taking into account authors Lustig and Xu, we suggest that family atmosphere significantly positively predicts work volition (Hypothesis 11). Additionally, some researchers found that family atmosphere significantly affected career adaptability. For instance, Zhao found that students living in a harmonious family atmosphere had the highest level of career adaptation, while students living in a frequently quarreling family atmosphere had the lowest level of adaptation [24]. Based on author Zhao, we suggest that family atmosphere positively predicts career adaptability (Hypothesis 12). Based on the findings of Wang et al. that family support can relieve personal stress so that individuals can have more energy to devote to their work [25], we suggest that family atmosphere significantly positively predicts future decent work perception (Hypothesis 13). In conjunction with the previous discussion regarding the relationships between family atmosphere, work volition, career adaptability, and future decent work perception, we assume that work volition mediates the association between family atmosphere and career adaptability (Hypothesis 14), as well as the association between family atmosphere and future decent work perception (Hypothesis 15), and that career adaptability mediates the association between family atmosphere and future decent work perception (Hypothesis 16).

School climate is a multi-level complex concept, which reflects the relatively lasting environmental characteristics of the school in terms of organizational structure, teaching practice, rules, goals, values, and interpersonal relationships [26]. It includes teacher support, peer support, and autonomy support [27]. Some previous studies have found that school climate significantly affects work volition, career adaptability, and perceptions of future decent work. For instance, Wang indicated that school climate has a stronger impact on newcomers’ career adaptability among Chinese students [28]. Therefore, based on author Wang, we suggest that school climate significantly positively predicts work volition (Hypothesis 17) and career adaptability (Hypothesis 18). Masdonati et al. (2019) indicated that a good climate can facilitate smooth and inclusive transitions from school to work, such that succeeding in this transition implies not only finding a job when leaving school but also accessing decent and dignified work [29]. Thus, we suppose that school climate significantly positively predicts future decent work perception (Hypothesis 19). In conjunction with the previous discussion regarding the relationships between school climate, work volition, career adaptability, and future decent work perception, we assume that work volition mediates the association between school climate and career adaptability (Hypothesis 20), and the association between school climate and future decent work perception (Hypothesis 21), while career adaptability mediates the association between school climate and future decent work perception (Hypothesis 22).

### 1.3. The Current Study

Rural college students were chosen as participants, because they are still in a relatively weak position, although they show stronger job-hunting initiative in the Chinese job market [30]. It is worth noting that rural college students in employment have a particular set of characteristics. First, most rural college students’ families belong to the lowest level of social stratification and lack wide network resources, which means relying on their own ability [31] and even fortune when hunting for jobs. Therefore, it is difficult for them to obtain abundant employment information and more employment opportunities. Second, due to the limitations of living conditions, they are lacking some essential abilities such as communication and organization, which leads to a lack of confidence when applying for a job.

**Figure 1:**
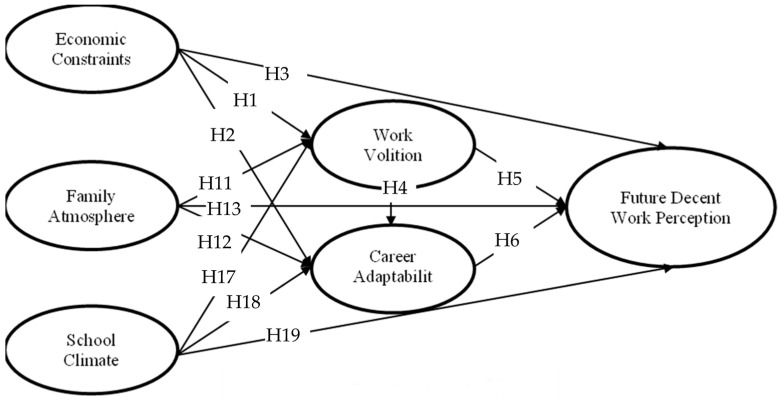
The theoretical model.

The aim of this study was to examine the 22 hypotheses, as mentioned above, about economic constraints, family atmosphere, school climate, work volition, career adaptability, and future decent work perception in rural college students (The entire model is shown in Figure 1).

## 2. Materials and Methods

### 2.1. Participants

We obtained 1100 datasets and omitted 154 datasets with poor response quality. In total, 946 complete datasets were acquired. The mean age of the respondents was 19.86 years (SD = 1.48). Participants identified as men (243, 25.7%) and women (703, 74.3%); 497 (52.5%) were in their first year of study, 249 (26.3%) in their second year, 119 (12.6%) in their third year, and 81 (8.6%) in their fourth year. Participants reported household incomes of less than RMB 1000 per month (n = 94, 9.9%), RMB 1000–3000 per month (n = 356, 37.6%), RMB 3000–6000 per month (n = 324, 34.2%), RMB 6000–10,000 per month (n = 124, 13.1%), RMB 10,000–30,000 per month (n = 39, 4.1%), and more than RMB 30,000 per month (n = 9, 1.0%). Detailed demographic information of the participants is shown in Table 1.

### 2.2. Procedure

Based on a convenience sampling method, this study first contacted several teachers of some schools, and then these teachers distributed the questionnaire links to college students with rural household registration. The Wenjuanxing (https://www.wjx.cn/vm/tUuoLXs.aspx (accessed on 20 December 2023)) online survey tool used in the current study is conducive to ensuring data collection without missing data. All participants were informed that their participation was anonymous and voluntary, and that they had the right to withdraw at any time. All participants completed these questionnaires, which took up to 15 min.

### 2.3. Ethics Statement

The study did not include minors. Informed consent was obtained from all subjects involved in the study. The participants provided consent by completing the questionnaire, and their participation was anonymized. The study was conducted in accordance with the Declaration of Helsinki and approved by the Ethics Committee of the School of Education, Minzu University of China (approval date: 1 March 2022).

### 2.4. Instruments

Economic constraints. Economic constraints were measured using the 5-item Economic Constraints Scale (ECS) [32]. Some items included “I considered myself very poor or very close to poverty for much of my life” and “For as long as I can remember I have had difficulties making ends meet. For most of my life, my financial situation was very precarious”. The students were asked to provide ratings based on a 7-point Likert scale ranging from 1 (strongly disagree) to 7 (strongly agree). In the current study, the estimated internal consistency of scales score was α = 0.93.

Family atmosphere. Family atmosphere was measured using the 8-item Family Atmosphere Subscale [21]. Items included “my family allows the family members to live in their own way” and “my parents allowed me to develop my interests and hobbies freely”. The students were asked to provide ratings based on a 5-point Likert scale ranging from 1 (strongly disagree) to 5 (strongly agree). In the current study, the estimated internal consistency of scales score was α = 0.79.

School climate. School climate was measured using the 25-item Perceived School Climate Scale [27]. The scale includes three dimensions, i.e., teacher support (containing 7 items such as “teachers in my school will help students with school problems”), peer support (containing 13 items such as “my classmates in my school will help each other”), and opportunities for autonomy (containing 5 items such as “students in my school have the opportunity to decide in class”). Based on the improvement of fit degree, we omitted some of the items in each dimension, leaving 7 items for teacher support, 7 items for peer support, and 5 items for opportunities for autonomy. The students were asked to provide ratings based on a 4-point Likert scale ranging from 1 (strongly disagree) to 4 (strongly agree). In the current study, the estimated internal consistency of scales score was α = 0.83.

Work volition. Work volition was measured using the 7-item Work Volition Scale [10]. Items included “I feel in full control of my future career choices.” The students were asked to provide ratings based on a 7-point Likert scale ranging from 1 (strongly disagree) to 7 (strongly agree). In the current study, the estimated internal consistency of scales score was α = 0.83.

Career adaptability. Career adaptability was measured using the Career Adaptability Scale [33,34]. The scale includes three dimensions: career concern (e.g., “I care more about my career”), career control (e.g., “I will overcome obstacles or I will fix the problem”), career curiosity (e.g., “I will be very curious about the new opportunities”), and career confidence (e.g., “I will stay optimistic”). The students were asked to provide ratings based on a 5-point Likert scale ranging from 1 (strongly disagree) to 5 (strongly agree). In the current study, the estimated internal consistency of scales score was α = 0.97.

Future decent work perceptions. Future decent work perception was measured using the 15-item Work Volition Scale [10]. Items included “I will get very good health care benefits from my work.” The students were asked to provide ratings based on a 5-point Likert scale ranging from 1 (strongly disagree) to 5 (strongly agree). In the current study, the estimated internal consistency of scales score was α = 0.79.

The scores for all scales were calculated using the arithmetic mean formula. Moreover, with Brislin’s back-translation procedures [35], parts of the scales were revised to the Chinese versions.

## 3. Results

### 3.1. Preliminary Analyses

We examined skewness and kurtosis before testing the structural model. The skewness and kurtosis of all variables were lower than |1.91| and |2.41|, respectively, denoting that the shape of the data distribution in the study may not be severely nonnormal because of the absolute values of skewness ≤ 3.0 and kurtosis ≤ 10.0. The highest VIF value was 1.93, which was far below the threshold of 10, denoting that collinearity was not a concern in the model [36]. To justify the limitation of method bias, Harman’s single factor test for common method variance was conducted with the help of SPSS. The result explains that the maximum variance of a single factor is 27.39%, which is below the limit of 50% [37]. Moreover, discriminant validity was used to test the extent to which a scale differs from other scales, with the square root of AVE on the diagonal of the scale mostly higher than the Pearson correlation between scales, which indicates that these scales have high discriminant validity.

Table 2 shows all the correlations between the six factors, means, and standard deviations.

### 3.2. Model Testing

We used structural equation modeling (SEM) in Mplus 8.3 with maximum likelihood estimation to test our models. The following model-to-data fit indices were chosen: RMSEA, comparative fit index (CFI), Tucker–Lewis index (TLI), root mean square error of approximation (RMSEA), and standardized root mean residual (SRMR). The thresholds were used as good model-fit indicators, which were set to RMSEA ≤ 0.10, TLI ≥ 0.90, CFI ≥ 0.90, and SRMR ≤ 0.10.

### 3.3. Measurement Model

We first constructed a measurement model to examine the correlations between latent variables and to examine the goodness of fit of the observed indicators on their associated latent constructs. This measurement model was a good fit to the data: χ^2^ (2739) = 7517.49, *p* < 0.001, RMSEA = 0.04, 90% CI [0.042, 0.044], CFI = 0.91, TLI = 0.90, and SRMR = 0.07. As such, we proceeded with testing our structural models. Factor correlations and descriptive statistics can be found in Table 2.

### 3.4. Hypothesized Structural Model

Our hypothesized structural model consisted of all hypothesized pathways; therefore, the model fit was the same as the measurement model. Family atmosphere had significant direct effects on work volition (β = 0.30, SE = 0.06, *p* < 0.001, 95% CI [0.20, 0.43]), career adaptability (β = 0.43, SE = 0.04, *p* < 0.001, 95% CI [0.27, 0.42]), and future decent work perception (β = 0.24, SE = 0.04, *p* < 0.001, 95% CI [0.10, 0.27]). Hypothesis 11, Hypothesis 12, and Hypothesis 13 were supported. School climate had significant direct effects on work volition (β = 0.32, SE = 0.12, *p* < 0.001, 95% CI [0.33, 0.82]), career adaptability (β = 0.13, SE = 0.07, *p* < 0.05, 95% CI [0.02, 0.30]), and future decent work perception (β = 0.11, SE = 0.07, *p* < 0.05, 95% CI [0.01, 0.29]). Hypothesis 17, Hypothesis 18, and Hypothesis 19 were supported. Work volition had significant direct effects on career adaptability (β = 0.29, SE = 0.04, *p* < 0.001, 95% CI [0.16, 0.31]) and future decent work perception (β = 0.58, SE = 0.06, *p* < 0.001, 95% CI [0.34, 0.56]). Hypothesis 4 and Hypothesis 5 were supported. However, economic constraints did not significantly predict work volition (*p* = 0.22, 95% CI [−0.01, 0.07]), career adaptability (*p* = 0.80, 95% CI [−0.02, 0.03]), or future decent work perception (*p* = 0.53, 95% CI [−0.04, 0.02]). Furthermore, career adaptability had no significant direct effects on future decent work perception (*p* = 0.32, 95% CI [−0.05, 0.14]). Hypothesis 1, Hypothesis 2, Hypothesis 3, and Hypothesis 6 were rejected.

### 3.5. Indirect Effects

Family atmosphere and school climate had significant indirect effects on future decent work perceptions via work volition (β = 0.18, SE = 0.03, *p* < 0.001, 95% CI [0.09, 0.20]; β = 0.19, SE = 0.06, *p* < 0.001, 95% CI [0.15, 0.37], respectively). Hypothesis 15 and Hypothesis 21 were supported. Family atmosphere and school climate had significant indirect effects on career adaptability via work volition (β = 0.09, SE = 0.02, *p* < 0.001, 95% CI [0.05, 0.11]; β = 0.09, SE = 0.04, *p* < 0.001, 95% CI [0.07, 0.21], respectively). Hypothesis 14 and Hypothesis 20 were supported. However, other indirect effects were not significant (see Table 3). Hypothesis 7, Hypothesis 8, Hypothesis 9, Hypothesis 10, Hypothesis 16, and Hypothesis 22 were rejected. The validation outcomes for all hypotheses are presented in Table 4.

## 4. Discussion

The goal of the current study was to examine the relationships between economic constraints, family atmosphere, and school climate, and work volition, career adaptability, and future decent work perception for rural college students. We found partial support for our hypotheses; the following text will provide a detailed explanation of the research results.

### 4.1. The Direct Relationship Between Several Variables

The unanticipated finding of this study is that economic constraints did not significantly predict work volition, career adaptability, and future decent work perceptions, which was not consistent with some previous studies [11,13,17]. There are several possible explanations for these results. First, to ensure that children fully concentrate on their studies without any feelings of inferiority or arrogance caused by family income, Chinese parents may tell a “white lie” to their children and hide their family’s real economic situation. Therefore, many students are ambiguous about their financial conditions, which may influence their judgment of economic constraints. Second, universities admit students based on their performance in the college entrance examination (gaokao) rather than their economic conditions in China. Many rural students, through their diligent studies, are able to be admitted to high-quality universities. Here, regardless of their economic level, these students can enjoy equal-quality vocational-related curricula, training, information consultation, internship opportunities, and other resources. Therefore, the impact of economic constraints on vocational outcomes is continuously diminished.

Similarly to the results of previous studies [15,38], our results support the effect of work volition on career adaptability and future decent work perceptions, indicating that work volition plays a crucial role in students’ career adaptability and future decent work perceptions. The primary reason for this may be that when individuals believe that they have the ability to overcome challenges and make free career choices, they are more likely to take proactive actions and improve their preparation for a certain profession. Moreover, this positive belief and mindset will drive individuals to make adjustments both mentally and practically, increasing their chances of obtaining decent jobs.

There was also a non-significant path from career adaptability to future decent work perceptions, which is inconsistent with the previous research conducted on Chinese nursing college students [13,14]. One possible explanation for this result is that there are a huge number of new graduates in the Chinese job market every year, including numerous excellent college students from various majors, which leads to an imbalance between the large population and the small number of decent recruitment positions. In this situation, whether an individual can get a decent job does not only depend on an individual’s level of preparation, but also the relative difference in preparation compared to others. That is to say, even if someone is highly prepared, there are chances that others are even more prepared; therefore, it may still be difficult for the former to get the position.

Our findings demonstrate that family atmosphere has a significant positive effect on work volition, career adaptability, and future decent work perceptions. Specifically, compared with those who do not possess the benefit of a good family atmosphere, those people who live in a good family environment are more likely to feel that they have a choice in their career decision-making and are more adaptable to jobs and securing decent work. The primary reason for this may be that a good family atmosphere provides powerful family support, including both instrumental and affective support [39]. To be specific, a beneficial family atmosphere is conducive to communication between family members on a variety of topics, including parents’ willingness to share their career experiences and advice with children, which unconsciously equips children with common sense for future work such as promotion, job-hopping, and workplace relationships [40]; the above information will make these children more confident about securing decent work, more adaptable when entering the workplace, and more likely to obtain decent work.

Our findings revealed that school climate has a significant positive effect on work volition, career adaptability, and future decent work perceptions. To elaborate, students with more friendly teacher-student relationships, close classmate relationships, and a reasonable school system tend towards better vocational outcomes. This is in line with the conclusions of previous studies that school climate factors significantly predicted career expectations and career intentions [41]. When students receive encouragement, praise, and respect from teachers, they no longer feel anxious or even fearful about oppression from authority figures. Thus, they are more willing to ask teachers about career-related issues and follow some beneficial advice according to their own needs. In turn, teachers working in a positive school climate have a stronger professional identity [42], which means they are full of enthusiasm and passion for students’ development, such as occupational planning, and would like to create more opportunities for students to explore their futures. They will strengthen students’ self-efficacy [43], career adaptability, and ability to perceive future decent work.

### 4.2. The Mediating Role of Work Volition and Career Adaptability

Work volition was a significant mediator linking family atmosphere and school climate to career adaptability and future decent work perceptions. Specifically, this finding demonstrates that family atmosphere and school climate can influence career adaptability and future decent work perceptions through work volition, which confirmed the results of previous research [38]. These mediation findings suggest that a supportive family and school environment enables students to be more optimistic and better prepared for their careers, which makes them feel confident to make work decisions. Additionally, when equipped with strong work volition, they generally tend to perceive that they have more control over the job search process and are more confident to obtain a career that they desire [44]. However, our findings revealed that work volition is not a mediator connecting economic constraints to career adaptability and future decent work perceptions, which is consistent with some previous studies [13]. As mentioned earlier, this article attributes this result to the survey tools used and the criteria of Chinese universities, which admit students based on their scores on the gaokao rather than their economic background.

Contrary to our expectations, career adaptability failed to play a mediating role in the relationship between economic constraint, family atmosphere, school climate, and work volition and future decent work perceptions, which is not supported by some studies [13]. A possible explanation for this limited finding may be that, for college students, getting a decent job not only relies on their own career ability, but also is always influenced by the effects of intense competition from their peer group, as mentioned above. Moreover, some scholars have also reflected on the role of adaptability in the theory; for example, Duffy et al. believe that other model variables account for most of the unique variances, but adaptability is not very applicable to the prediction of decent work [15].

### 4.3. Practical and Theoretical Implications

For Chinese rural college students, the main goal of higher education is to get decent work and then change their destinies [45]. However, a unique structure of rural settings with limited available resources [46] may make the process more difficult. The findings in this study are of some significance in improving future decent work perception for these students. In order to prepare this striving group to obtain meaningful and satisfying work more easily, more powerful support from diverse people and vocational interventions should be implemented. First, our findings suggest that the family atmosphere is helpful in improving a student’s career life. Family members should increase the amount of time they spend with each other, respect and support individual ideas, and form a good family environment together. Second, the career development of college students is also dependent on the school climate. In fact, since the successful implementation of school climate interventions is largely dependent on staff members [47] and whole-school investment [48,49,50], it may be a necessary prerequisite that staff members should take encouraging and inclusive educational methods to make students feel as warm as possible in the school atmosphere. Third, the mediating effects and the malleable nature of work volition may inform possible interventions. It may be beneficial for counselors to assess clients’ levels of work volition by exploring barriers that limit clients’ possible career options [51]. Then, counselors may make efforts to break down those difficulties into small problems that can be easily resolved and organize a list of action plans to guide clients to overcome them. In particular, when working with clients with low levels of work volition, counselors are suggested to help them to find inspirational cases that had similar constraints as theirs but secured a satisfying job, in order to encourage clients to learn from successful experiences.

Additionally, this study holds significant theoretical implications. First, it reveals that some previous conclusions are indeed valid among Chinese subjects, while others do not hold true, thereby providing a critical test of Psychology of Working Theory. Second, this research introduces two new variables—family atmosphere and school climate—and identifies their close relationships with existing variables in work psychology. This is of considerable importance, as it not only expands the scope of the Psychology of Working Theory, but also enriches the knowledge framework within the field of vocational psychology.

### 4.4. Limitations and Future Directions

This study has several limitations which could provide directions for future research. First, the research adopted a cross-sectional study design, which means casual relationships between variables in the study are difficult to confirm. In order to further ascertain the causal mechanisms of these variables and explore the dynamic career trajectories of Chinese rural college students, future studies should utilize a longitudinal research design, obtaining data at multiple time points and implementing experimental methods. Second, participants in this study were from various rural family backgrounds. Data from these students with advanced conditions in all aspects and nearly without difference from cities may partly obscure some information provided by those truly rural disadvantaged students, which leads to some possible variation in our results. Thus, future studies should obtain more representative samples from rural family backgrounds in line with the concept of vulnerable groups. Third, the participants of the present study are a very particular population, such that special attention should be paid to the generalization of the results of this study. Therefore, future studies should focus on recruiting diverse participant groups to validate and expand upon the findings of this study.

## 5. Conclusions

The current study provides support for the relationship between economic constraints, family atmosphere, and school climate and work volition, career adaptability, and future decent work perception. Although this study has limitations, it informs future studies by highlighting the important role of family atmosphere, school climate, and work volition. Specifically, both the family atmosphere and school climate play a positive role in enhancing individuals’ work volition and career adaptability as well as future decent work perception. Furthermore, work volition also significantly contributes to career adaptability as well as future decent work perception. Meanwhile, these findings also provide practical implications for families and schools to create a positive atmosphere for rural college students and improve their work volition.

## Figures and Tables

**Table 1:** Demographic information of the participants.

Demographic Variable		Sample	
		Number	Percentage
Gender	men	243	25.7%
	women	703	74.3%
Grade	freshman	497	52.5%
	sophomore	249	26.3%
	junior	119	12.6%
	senior	81	8.6%
Household incomes (per month)	less than 1000 RMB	94	9.9%
	1000–3000 RMB	356	37.6%
	3000–6000 RMB	324	34.2%
	6000–10,000 RMB	124	13.1%
	10,000–30,000 RMB	39	4.1%
	>30,000 RMB	9	1.0%

**Table 2:** Descriptive Statistics and Correlations.

	M	SD	EC	FA	SC	WV	CA	FDWP
EC	4.37	1.34	0.88					
FA	3.63	0.57	−0.06	0.63				
SC	2.99	0.39	−0.06	0.39 ***	0.55			
WV	4.51	0.95	0.07 *	0.37 ***	0.28 ***	0.66		
CA	4.00	0.60	0.02	0.58 ***	0.44 ***	0.53 ***	0.73	
FDWP	3.30	0.44	−0.13 ***	0.51 ***	0.37 ***	0.53 ***	0.51 ***	0.51

Note. EC = economic constraints, FA = family atmosphere, SC = school climate, WV = work volition, CA = career adaptability, FDWP = future decent work perceptions. * *p* < 0.05, *** *p* < 0.001

**Table 3:** Indirect effects from the structural model.

Paths		β	SE	95% Confidence Interval Bootstrap Bias Corrected	
				Lower Bound	Upper Bound
EC→FDWP	Total effect	0.01	0.02	−0.03	0.03
	Indirect effect	0.03	0.01	−0.01	0.03
	EC→WV→FDWP	0.03	0.01	−0.01	0.03
	EC→CA→FDWP	0	0.001	−0.001	0.002
	EC→WV→CA→FDWP	0.001	0	0.00	0.002
	Direct effect	−0.02	0.01	−0.04	0.02
FA→FDWP	Total effect	0.44 ***	0.04	0.26	0.43
	Indirect effect	0.20 ***	0.03	0.09	0.23
	FAA→WV→FDWP	0.18 ***	0.03	0.09	0.20
	FAA→CA→FDWP	0.02	0.02	−0.01	0.05
	FAA→WV→CA→FDWP	0.004	0.003	−0.003	0.01
	Direct effect	0.24 ***	0.04	0.10	0.27
SC→FDWP	Total effect	0.30 ***	0.09	0.24	0.58
	Indirect effect	0.20 ***	0.06	0.16	0.39
	SC→WV→FDWP	0.19 ***	0.06	0.15	0.37
	SC→CA→FDWP	0.01	0.01	−0.01	0.03
	SC→WV→CA→FDWP	0.01	0.01	−0.01	0.02
	Direct effect	0.11 *	0.07	0.01	0.29
WV→FDWP	Total effect	0.60 ***	0.06	0.35	0.57
	Indirect effect (WV→CA→FDWP)	0.01	0.01	−0.01	0.03
	Direct effect	0.58 ***	0.06	0.34	0.56
EC→CA	Total effect	0.02	0.01	−0.02	0.03
	Indirect effect (EC→WV→CA)	0.01	0.01	−0.001	0.02
	Direct effect	0.01	0.01	−0.02	0.03
FA→CA	Total effect	0.52 ***	0.04	0.34	0.51
	Indirect effect (FAA→WV→CA)	0.09 ***	0.02	0.05	0.11
	Direct effect	0.43 ***	0.04	0.27	0.42
SC→CA	Total effect	0.22 ***	0.08	0.15	0.44
	Indirect effect (SC→WV→CA)	0.09 ***	0.04	0.07	0.21
	Direct effect	0.13 **	0.07	0.02	0.30

Note. EC = economic constraints, FA = family atmosphere, SC = school climate, WV = work volition, CA = career adaptability, FDWP = future decent work perceptions. * *p* < 0.05, ** *p* < 0.01, *** *p* < 0.001.

**Table 4:** Validation results of the hypotheses.

Hypothesis	Hypothesis Path	Result	Hypothesis	Hypothesis Path	Result
H1	EC→WV	Rejected	H12	FA→CA	Supported
H2	EC→CA	Rejected	H13	FA→FDWP	Supported
H3	EC→FDWP	Rejected	H14	FA→WV→CA	Supported
H4	WV→CA	Supported	H15	FA→WV→FDWP	Supported
H5	WV→FDWP	Supported	H16	FA→CA→FDWP	Rejected
H6	CA→FDWP	Rejected	H17	SC→WV	Supported
H7	EC→WV→CA	Rejected	H18	SC→CA	Supported
H8	EC→WV→FDWP	Rejected	H19	SC→FDWP	Supported
H9	EC→CA→FDWP	Rejected	H20	SC→WV→CA	Supported
H10	WV→CA→FDWP	Rejected	H21	SC→WV→FDWP	Supported
H11	FA→WV	Supported	H22	SC→CA→FDWP	Rejected

Note. EC = economic constraints, FA = family atmosphere, SC = school climate, WV = work volition, CA = career adaptability, FDWP = future decent work perceptions.

## Data Availability

Data supporting reported results are available from the authors on request.
